# Pre-operative embolization of a complex systemic to pulmonary vascular malformation

**DOI:** 10.1259/bjrcr.20230056

**Published:** 2023-09-11

**Authors:** Michael Brassil, Yangmei Li, Michael Ko, Marie E Faughnan, Vikramaditya Prabhudesai

**Affiliations:** 1 Department of Medical Imaging, St Michael’s Hospital, Unity Health Toronto, Toronto, Canada; 2 Department of Thoracic Surgery, St Joseph’s Health Center, Unity Health Toronto, Toronto, Canada; 3 Department of Medicine, Division of Respirology, St Michael’s Hospital and Li Ka Shing Knowledge Institute, University of Toronto, Toronto, Canada; 4 Li Ka Shing Knowledge Institute, St Michael’s Hospital, Toronto, Canada; 5 Toronto HHT Centre, St Michael’s Hospital, Toronto, Canada

## Abstract

A 38-year-old male patient presenting with mild exertional dyspnea was noted to have a lingular opacity on chest radiograph. CT of the chest demonstrated an unusual complex inferior lingular vascular malformation with branches arising from the left internal thoracic artery and the left inferior diaphragmatic artery via the celiac artery. There was suspected communication with both pulmonary arterial and venous branches. Following thorough assessment and comprehensive clinical investigation, the patient elected to proceed to definitive surgical management due to potential risk of life-threatening hemoptysis. Interventional radiology performed pre-operative diagnostic angiography and embolization of the systemic feeding arteries. The patient proceeded to have an uncomplicated video-assisted thoracoscopic surgery segmentectomy and was discharged the next day. The patient was asymptomatic at follow-up with complete resolution of the malformation on CT at 6 months. We discuss an uncommon pathology which benefited from multidisciplinary management including successful pre-operative endovascular embolization.

## Introduction

**Figure 1.**
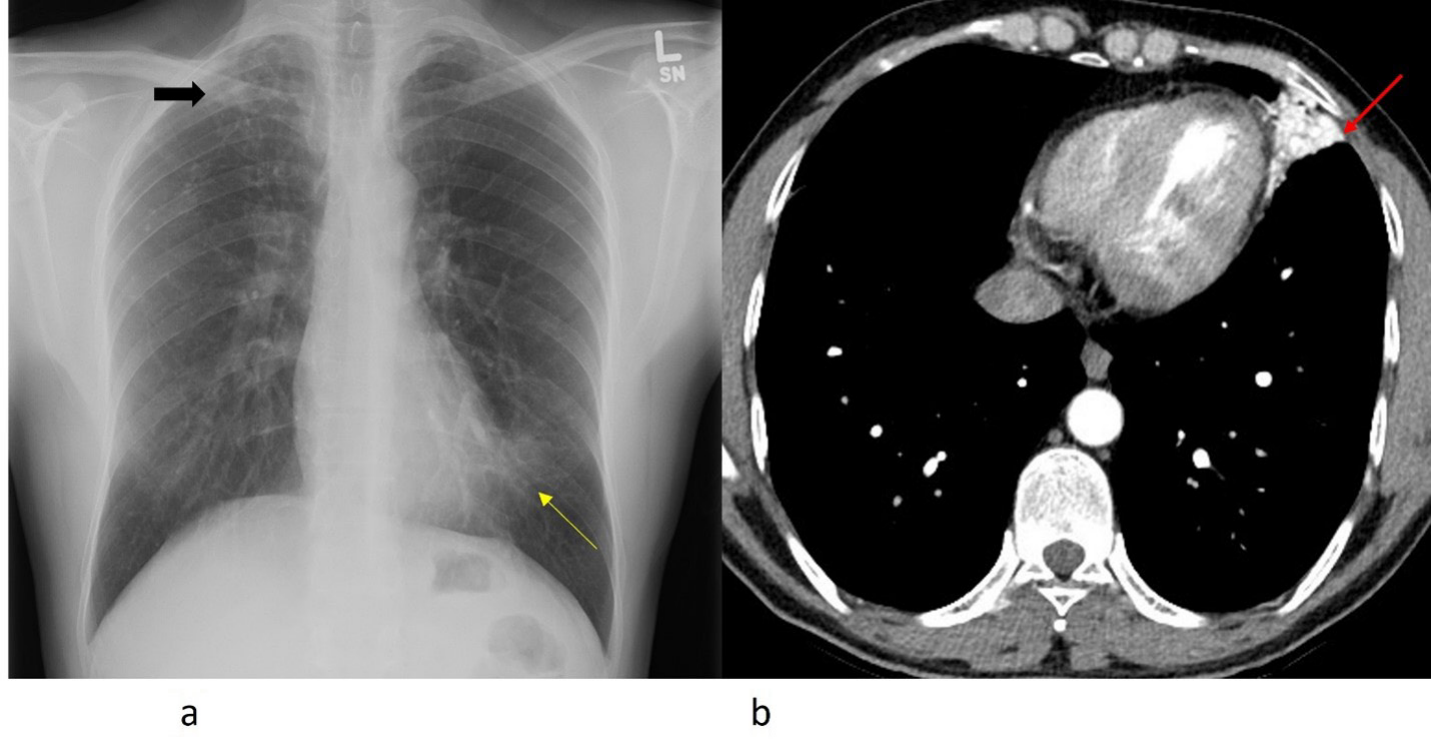
(**a**) Chest radiograph demonstrating right upper lobe fibrocalcific scarring associated with prior TB infection (Black arrow) and a faint lingular opacity obscuring the left heart border (yellow arrow). (**b**) Axial contrast-enhanced image of the mid-thorax demonstrating lingular vascular malformation with numerous tortuous dilated vessels (Red arrow). TB, tuberculosis.

A 38-year-old male patient presented to his family physician with a 1-year history of exertional dyspnea which had worsened in the previous 6 weeks. The patient had immigrated to Canada from East Africa 2 years previously and had been treated for tuberculosis for 6 months prior to arrival based on an abnormal immigration screening chest X-ray. Subsequent bronchoscopy and bronchioalveolar lavage were negative for TB. A repeat chest X-ray in St Michael's Hospital, Unity Health Toronto (Figure 1a) was noted to have right upper lobe fibrocalcific changes typical of previous TB infection and a lingular opacity. CT of the chest was performed (Figure 1b) which demonstrated an unusual complex inferior lingular vascular malformation with branches arising from the left internal thoracic artery and the left inferior diaphragmatic artery via the celiac artery. There was suspected communication with both pulmonary arterial and venous branches.

The patient had no personal history of hemoptysis but had one episode of left anterior pleuritic chest pain 1 year ago and felt unwell for 2 weeks. He didn’t seek medical attention at that time, as it was the beginning of the COVID-19 pandemic, and he was reluctant to leave home. He has a medical history of dyslipidemia which is well controlled with statin therapy. He is a current smoker with a 20 pack-year smoking history. His physical examination and hematological investigations were normal. There was no evidence of any other vascular malformations or any associated congenital or familial disease.

The patient was assessed by respiratory medicine, thoracic surgery and interventional radiology (IR), for risk of hemorrhage and discussions on options for management. Options discussed included conservative management (clinical and imaging surveillance), embolization by IR or surgical resection. Ultimately, the patient’s preference was for definitive surgical management and following multidisciplinary discussion the decision was made for IR to embolize the vascular malformation followed by surgical resection. The rationale was to better delineate the vascular anatomy and to reduce the risk of intraoperative hemorrhage.

## Technique

**Figure 2.**
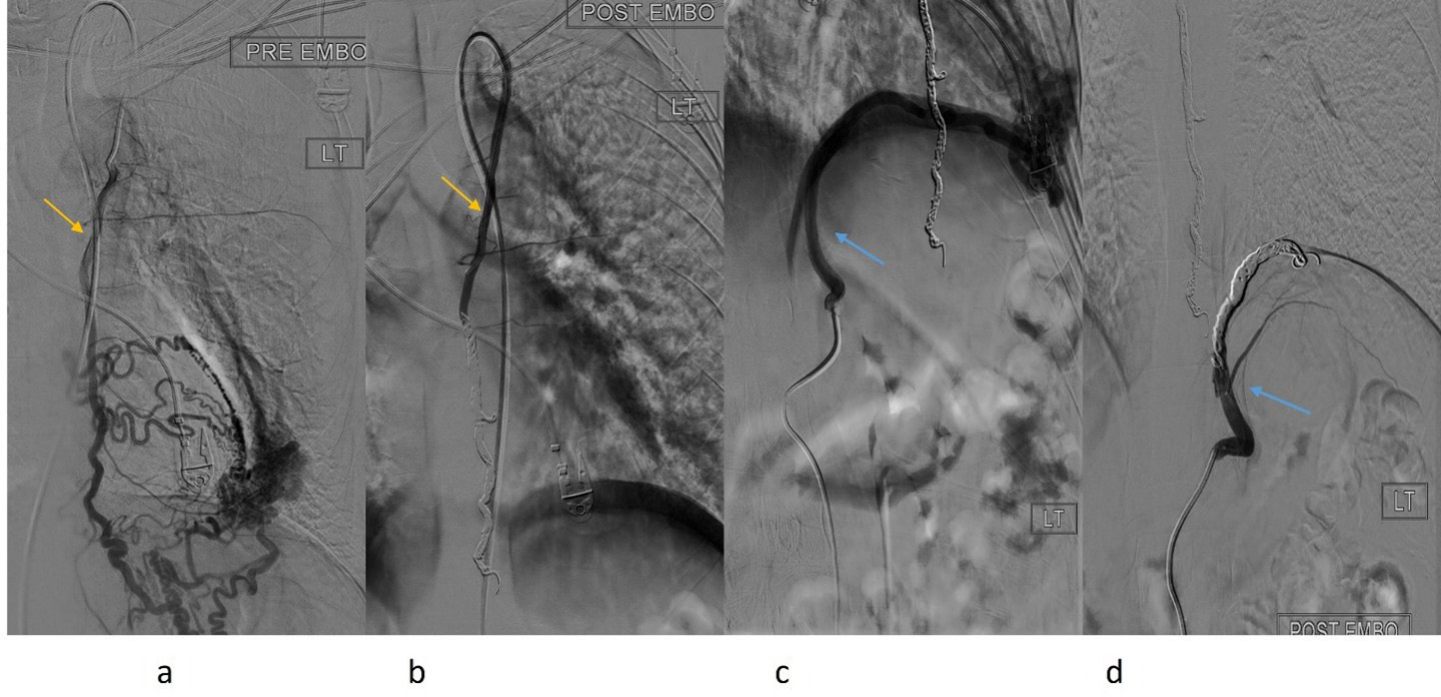
Catheter-directed digital subtracted angiography of the left internal thoracic artery pre- and post-embolization (yellow arrow, **a, b**) and the left inferior diaphragmatic artery pre- and post-embolization (blue arrow, **c, d**).

The patient first presented to IR for single session diagnostic angiography and embolization. They were positioned supine on the angiography table and under ultrasound guidance, the right common femoral artery was punctured in a retrograde fashion and a 5 French (Fr) vascular sheath was placed and connected to continuous saline irrigation. A 5-Fr Bernstein catheter and 0.035-inch hydrophilic guidewire (Terumo) combination were introduced through the sheath and access to the left subclavian artery and subsequently, the left internal thoracic artery was achieved. A selective angiogram was performed (Figure 2a) which demonstrated multiple aberrant arterial branches arising from the mid to distal left internal thoracic artery and supplying the nidus of a complex vascular malformation in the lingula. There was angiographic evidence of shunting to branches of the lingular pulmonary artery and tributaries of the pulmonary vein and for this reason liquid embolization could not be performed. A 2.8-Fr microcatheter and microwire (Progreat, Terumo) was introduced through the Bernstein catheter and advanced distally in the internal thoracic artery to the level of the diaphragm where there was some resistance and contrast injection demonstrated a localized arterial dissection. IR then proceeded to embolize from this point proximally using a 6 mm pushable framing coil (Nester, Cook Medical) as a backstop, followed by multiple packing coils (Penumbra) which mechanically occluded the distal left internal thoracic artery (Figure 2b). Repeat selective angiogram through the Bernstein catheter demonstrated satisfactory occlusion with no additional supply to the vascular malformation from the left internal thoracic artery. Next, the Bernstein catheter was exchanged for a 5-Fr Simmons one catheter which was formed and used to select the coeliac trunk. Selective angiography in multiple obliquities was performed which demonstrated the origin of a large arterial feeding vessel arising directly from the coeliac trunk and supplying the lingular vascular malformation (Figure 2c). The ostium of this artery (the left inferior phrenic artery) was selected with the Simmons catheter and the same microcatheter system was used to advance more distally. Once again, there was resistance to advancement of the microwire at the level of the diaphragm and the vessel was embolized from this point using detachable framing coil (POD6, Penumbra) and packing coils (Penumbra). Repeat selective angiogram via the base catheter demonstrated excellent result with cessation in antegrade flow within the feeding vessel (Figure 2d). IR decided to conclude the procedure at this point. We felt that the malformation had been sufficiently devascularized to facilitate surgical resection and further selective angiography and embolization from pulmonary arteries was unlikely to confer additional benefit. If the patient was not for definitive surgical management, we may have pursued this approach to reduce the likelihood of collateral reperfusion. The catheters and wires were removed. Puncture site hemostasis was achieved using 6-Fr Perclose closure device (Abbott Vascular, CA, USA). There were no immediate complications, and the patient was discharged later that day following a period of bedrest and observation.

**Figure 3.**
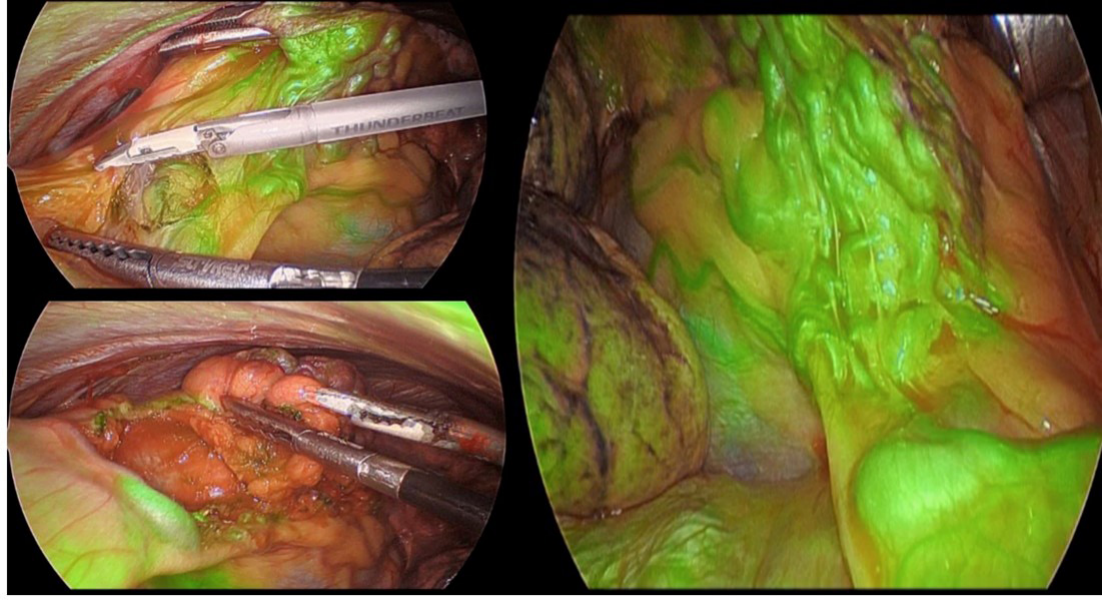
Intraoperative photographs of the large number of aberrant vessels coursing through the pericardial fat pad, being highlighted by ICG dye(an *in vivo* fluorescent dye that highlights the blood vessels) without the presence of the dye it would be very difficult to identify where these are as they are hidden within the fat; this helps with direct visualization for ligation with the harmonic scalpel. ICG, Indocyanine green.

The following week, the patient was admitted for surgical resection, as planned. The decision was made to proceed to video-assisted thoracoscopic surgery (VATS) and lingulectomy. After induction of general anesthesia and single lung ventilation, general thoracoscopy was carried out. There were numerous vessels feeding the nidus in the lingula which was densely consolidated with aberrant blood vessels. These blood vessels coursed from the diaphragm through a very large branch, as well as branches of the pericardiophrenic artery and the internal mammary artery. Additional ports were then placed. The inferior pulmonary ligament was taken down using electrocautery. At this point, Indocyanine green (ICG) dye was administered to the patient. Under direct thoracoscopic evaluation, each individual branch going into the lingula was visualized. This was seen coursing through the fat as fluorescent green vessels (Figure 3). The Thunderbeat device was then used to transect each of these branches individually. Care was taken not to damage the phrenic nerve during this dissection. Each branch was isolated, and the combination of electric and ultrasonic coagulation was employed to seal each vessel. The larger vessels were also clipped. The malformation was dissected off the pericardium and the phrenic nerve was skeletonized. After control of the artery and vein, the entire lingula was dissected was resected en-bloc using 3 firings of the GIA stapler. The specimen was then placed in an Endo Catch bag and brought out through the anterior utility incision. A 28-Fr chest tube was inserted and directed superiorly towards the apex. The patient was extubated in the operating room and brought to the recovery in stable condition. The patient was discharged home on post-operative Day 1.

## Discussion

Pulmonary vascular malformations are a heterogenous group of conditions, whereby there is an abnormal capillary-free communication between blood vessels in the chest. The most common pulmonary vascular malformation is pulmonary arteriovenous malformations (PAVMs) which may occur as frequently in approximately 1 in 1600 patients.^1^PAVMs are associated with hereditary hemorrhagic telangiectasia (HHT) in 80% of cases, and PAVMs are present in 40% of patients with HHT. PAVMs are direct connections between a pulmonary artery and pulmonary vein, with no intervening pulmonary capillaries. This leads most frequently to neurological complications, such as stroke or brain abscess, and less frequently to hemorrhage, hypoxemia and shortness of breath.^2^ Preventative embolization of PAVMs is recommended, in HHT patients and idiopathic cases.^3^

Systemic to pulmonary vascular malformations are a less common group of conditions which may be congenital or acquired. Our knowledge of these malformations is mainly derived from published case reports, where various vascular anatomical variations have been described. A systematic review of case reports of internal mammary artery to pulmonary vascular fistulae reviewed 80 case reports on the condition. The majority of cases (47) were iatrogenic related to coronary artery bypass surgery, 12 were deemed to be congenital while the remainder (21) were associated with inflammatory, infectious or malignant disease, of these TB was implicated in four cases.^4^ The risk of adverse outcomes with these malformations is less clear. These cases were managed surgically (24%), medically (31%), with endovascular coiling (12%) or endovascularly with a polytetrafluoroethylene stent (10%).

In addition, there have been many reports of congenital bronchial artery to pulmonary vascular malformations where presentations are incidental and asymptomatic or with hemoptysis. These have been managed successfully with endovascular coiling^5^ or with attempted endovascular coiling and subsequent VATS resection.^6^

A case presentation similar to ours was reported by Geyik et al.^7^ where an asymptomatic patient was found to have a malformation involving the lingular pulmonary artery and supplied by both the left internal thoracic artery and the left phrenic artery.^7^ The authors treated the malformation by distal embolization of feeding systemic arteries using N-butylcyanoacrylate and distal embolization of the draining pulmonary artery using multiple large coil springs as well as a detachable balloon in order to avoid possible complications for the patient such as pulmonary hypertension and hemorrhage.

In our case report, the etiology of the complex systemic to pulmonary malformation is most likely congenital as the parenchymal fibrocalcific scarring from prior TB is remote to the malformation and likely not associated. The management was guided by the patient’s desire for definitive surgical resection to avoid theoretical risk of massive hemoptysis. Due to the complexity of the case, a multidisciplinary approach was taken to first achieve better anatomical understanding through digital subtracted angiography and then to embolize the main feeding arteries to minimize the risk of intraoperative hemorrhage. Our choice of embolic device was influenced by many factors including the flow rate and potential of left to right shunting. The caliber and tortuosity of the feeding systemic arteries prevented deployment of vascular plugs, which we would routinely use in PAVM embolization, often in combination with detachable coils. The choice of embolic agent is ever expanding with development of detachable coils, vascular plugs and microvascular plugs which are considered safe and effective in treatment of PAVM as outlined in a recently published summary of literature on PAVM embolization outcomes of various embolic devices published in the last 10 years.^8^ The use of ICG dye to highlight the numerous vessels which were otherwise buried in the pericardial fat was also a novel use of intraoperative imaging. Although AVM visualization using ICG has been well described in neurosurgical applications, the use in thoracic surgery has not yet been described.^9^ We believe our novel multidisciplinary approach to treating an exceedingly rare systemic to pulmonary vascular malformation resulted in an excellent outcome for our patient who benefited from combined minimally invasive definitive management.

## References

[1] Nakayama M, Nawa T, Chonan T, Endo K, Morikawa S, Bando M, et al . Prevalence of pulmonary arteriovenous malformations as estimated by low-dose thoracic CT screening. Intern Med 2012; 51: 1677–81. doi: 10.2169/internalmedicine.51.7305 22790125

[2] Shovlin CL . Pulmonary arteriovenous malformations. Am J Respir Crit Care Med 2014; 190: 1217–28. doi: 10.1164/rccm.201407-1254CI 25420112 PMC4315816

[3] Faughnan ME, Mager JJ, Hetts SW, Palda VA, Ratjen F . Second international guidelines for the diagnosis and management of hereditary hemorrhagic telangiectasia. Ann Intern Med 2021; 174: 1035–36. doi: 10.7326/L21-0067 34280351

[4] Abdul Jabbar A, Patel A, Marzlin N, Altabaqchali S, Hasan M, Al-Zubaidi M, et al . Internal Mammary artery-to-pulmonary vasculature Fistula: systematic review of case reports. Vasc Med 2017; 22: 426–31. doi: 10.1177/1358863X17724262 28990495

[5] Yon JR, Ravenel JG . Congenital bronchial artery-pulmonary artery fistula in an adult. Journal of Computer Assisted Tomography 2010; 34: 418–20. doi: 10.1097/RCT.0b013e3181d1e96e 20498547

[6] VanDerPloeg DG, Strong WR, Krohmer SJ, O’Connor WN, Martin JT . Congenital bronchial artery to pulmonary artery fistula presenting as hemoptysis. Ann Thorac Surg 2015; 99: e19–20. doi: 10.1016/j.athoracsur.2014.10.057 25555983

[7] Geyik S, Yavuz K, Keller FS . Unusual systemic artery to pulmonary artery malformation without evidence of systemic disease, trauma or surgery. Cardiovasc Intervent Radiol 2006; 29: 897–901. doi: 10.1007/s00270-004-0289-9 16404502

[8] Majumdar S, McWilliams JP . Approach to pulmonary arteriovenous malformations: A comprehensive update. J Clin Med 2020; 9: 1–26: 1927. doi: 10.3390/jcm9061927 PMC7356967 32575535

[9] Foster CH, Morone PJ, Tomlinson SB, Cohen-Gadol AA . Application of indocyanine green during arteriovenous malformation surgery: Evidence, techniques, and practical pearls. Front Surg 2019; 6: 70. doi: 10.3389/fsurg.2019.00070 31921884 PMC6917574

